# Distinct senescence mechanisms restrain progression of dysplastic nevi

**DOI:** 10.1093/pnasnexus/pgae041

**Published:** 2024-02-15

**Authors:** Franziska K Lorbeer, Gabrielle Rieser, Aditya Goel, Meng Wang, Areum Oh, Iwei Yeh, Boris C Bastian, Dirk Hockemeyer

**Affiliations:** Department of Molecular and Cell Biology, University of California, Berkeley, Berkeley, CA 94720, USA; Department of Molecular and Cell Biology, University of California, Berkeley, Berkeley, CA 94720, USA; Department of Molecular and Cell Biology, University of California, Berkeley, Berkeley, CA 94720, USA; Department of Dermatology, University of California, San Francisco, San Francisco, CA 94143, USA; Rebus Biosystems, Santa Clara, CA 95050, USA; Department of Dermatology, University of California, San Francisco, San Francisco, CA 94143, USA; Department of Pathology, University of California, San Francisco, San Francisco, CA 94143, USA; Helen Diller Family Comprehensive Cancer Center, University of California, San Francisco, San Francisco, CA 94143, USA; Department of Dermatology, University of California, San Francisco, San Francisco, CA 94143, USA; Department of Pathology, University of California, San Francisco, San Francisco, CA 94143, USA; Helen Diller Family Comprehensive Cancer Center, University of California, San Francisco, San Francisco, CA 94143, USA; Department of Molecular and Cell Biology, University of California, Berkeley, Berkeley, CA 94720, USA; Chan Zuckerberg Biohub, San Francisco, CA 94158, USA; Innovative Genomics Institute, University of California, Berkeley, CA 94720, USA

**Keywords:** dysplastic nevi, melanoma, TERT promoter mutations, telomere length, senescence

## Abstract

Telomerase reverse transcriptase (TERT) promoter mutations (TPMs) are frequently found in different cancer types, including ∼70% of sun-exposed skin melanomas. In melanoma, TPMs are among the earliest mutations and can be present during the transition from nevus to melanoma. However, the specific factors that contribute to the selection of TPMs in certain nevi subsets are not well understood. To investigate this, we analyzed a group of dysplastic nevi (DN) by sequencing genes commonly mutated in melanocytic neoplasms. We examined the relationship between the identified mutations, patient age, telomere length, histological features, and the expression of p16. Our findings reveal that TPMs are more prevalent in DN from older patients and are associated with shorter telomeres. Importantly, these TPMs were not found in nevi with BRAF V600E mutations. Conversely, DN with BRAF V600E mutations were observed in younger patients, had longer telomeres and a higher proportion of p16-positive cells. This suggests that these nevi arrest growth independently of telomere shortening through a mechanism known as oncogene-induced senescence (OIS). These characteristics extend to melanoma-sequencing datasets, where melanomas with BRAF V600E mutations were more likely to have a *CDKN2A* inactivation, overriding OIS. In contrast, melanomas without BRAF V600E mutations showed a higher frequency of TPMs. Our data imply that TPMs are selected to bypass replicative senescence (RS) in cells that were not arrested by OIS. Overall, our results indicate that a subset of melanocytic neoplasms face constraints from RS, while others encounter OIS and RS. The order in which these barriers are overcome during progression to melanoma depends on the mutational context.

**Significance Statement**

Dysplastic nevi (DN) are risk indicators and potential precursors of melanoma. Currently, the genetic makeup and the factors that lead to the arrest or progression of DN are poorly understood. There is an unmet need to understand in which context the proliferation of melanocytes is restricted predominantly by oncogene-induced senescence (OIS) and under what circumstances telomere shortening prevents cancer progression. Therefore, we characterized mutations, telomere length, and p16 expression in a prospectively collected and histopathologically validated set of DN. We find that there are two primary mechanisms at work in the arrest of DN: DN with strong mitogen activated protein kinase (MAPK) signaling preferentially trigger OIS. In DN, where OIS is bypassed, telomere shortening eventually triggers replicative senescence.

## Introduction

Dysplastic melanocytic nevi are initiated by mutations in proto-oncogenes activating the mitogen activated protein kinase (MAPK) pathway and are a risk factor and potential precursor for melanoma (1, 2). The hyperphysiological activation of MAPK signaling triggers a wave of melanocyte cell divisions, which are then thought to be restrained from further proliferation and progression to melanoma by a process called oncogene-induced senescence (OIS) (3–6). The hyperactivation and fast proliferation lead to continuous DNA replication stress that can trigger cell cycle arrest and thereby incrementally deplete the pool of dividing cells, resulting in a stable lesion of growth-arrested cells (7, 8). It has been demonstrated that the two tumor suppressors, p53 and pRB, are the major regulators of OIS and that the loss of cell-cycle checkpoint genes, such as *TP53* or *CDKN2A*, can bypass this arrest (9–11). However, the strength with which individual mutations activate the MAPK pathways can differ. One of the most frequent and strongest mutations is BRAF V600E - in comparison to other mutations in BRAF or inactivating mutations, such as loss of NF1, which are considered to be only weaker activating mutations of MAPK signaling (12, 13). These differences result in pathophysiological and molecular differences in nevi with BRAF V600E compared with those with other driver mutations. For example, nevi with the BRAF V600E mutation are more frequently associated with distinct histological features, such as more nested intraepidermal melanocytes, larger junctional nests, abrupt lateral circumscription, and larger cell size (14).

Recent sequencing efforts have revealed that there are intermediate melanocytic neoplasms with driver mutations in addition to the nevus-initiating MAPK-activating mutations, which fall short of melanoma. For example, mutations in the promoter of telomerase reverse transcriptase (TERT) have been observed in such melanocytic tumors and thus can occur at the transition from nevus to melanoma, preceding the selection of mutations that deactivate cell-cycle checkpoints (11, 15). Telomerase is responsible for maintaining chromosome ends and is required for the continuous proliferation of most human tissue stem cells and highly long-term proliferative cell types (16, 17). The so-called TERT promoter mutations (TPMs), which subvert the transcriptional silencing of *TERT* (18, 19), are the most common noncoding mutations in cancer (20). In cells without telomerase expression, telomeres shorten continuously and due to the gradual erosion of telomeres, cells eventually enter replicative senescence (RS). Thus, by triggering RS, telomere shortening functions as a strong tumor suppressor pathway, which leads to permanent growth arrest (21). TPMs enable telomerase to be expressed and thereby provide a means for somatic cells to evade RS. TPMs become a selective advantage for cells whose telomeres are exhausted and have become limited in their proliferation (19).

As a subset of melanocytic nevi, dysplastic nevi (DN) have defining characteristic clinical and histopathologic features. They are larger than most common acquired nevi and display a characteristic immune and stromal response (22, 23). While the initiating driver mutations and additional mutations of melanomas have been well cataloged by sequencing studies (12, 24, 25), the mutational landscape of DN, their telomere length, and their senescence state have not been extensively characterized. The telomeres of telomerase-immortalized cancer cells are comparatively short, and initially TPMs are not sufficient to immortalize cells, as additional cellular changes are required to fully stabilize the chromosome ends (19). One such additional change was recently described, where telomerase activity at telomeres is increased by mutations in the promoter of the *ACD* gene, which encodes the telomerase interactive protein TPP1 (26). These findings highlight that our understanding of the events required for the immortalization of cells, and the physiological context in which TPMs undergo positive selection is still incomplete. By the analysis of small cohorts of melanocytic nevi, it has been shown that TPMs can already be found in melanocytic nevi (27); however, most comprehensive studies of genomic aberrations of melanocytic and DN did not investigate TPMs as they analyzed only the coding region of the genome (28).

The early occurrence of TPMs in some melanocytic nevi poses a central challenge to our current understanding of the genetic makeup of DN and their progression to melanoma. If all nevi were effectively restrained by OIS, their cells would not undergo enough cell divisions to reach RS, and thus not require TPMs to support their immortalization. To resolve the role of telomeres and TPMs in early melanoma development, it is necessary to determine the genetic context in which telomeres become exhausted.

## Results

### TPMs are found in DN of older patients and in a mutually exclusive manner with BRAF V600E

**Fig. 1.**
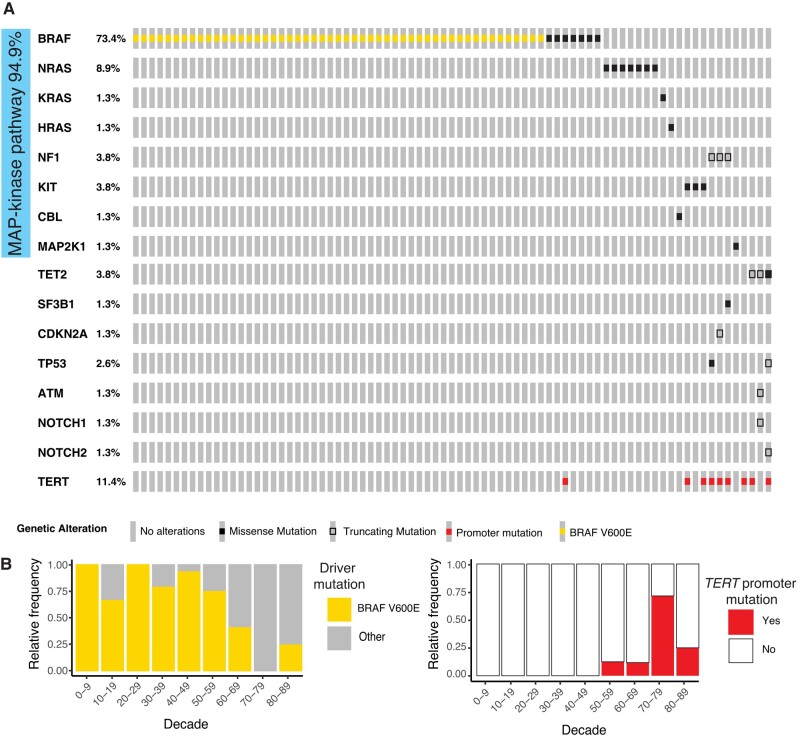
Somatic mutations in DN. BRAF V600E mutations are more common in younger patients where other mutations predominate in older patients, partially combined with *TERT* promoter mutations. A) Tiling plot of pathogenic and likely pathogenic mutations. Side bar indicates all mutations affecting the MAPK pathway. B) Relative frequency of BRAF V600E (left) and *TERT* promoter mutations (right) by age decade.

To elucidate the genetic context of DN in which TPMs are selected, we sequenced exons and noncoding regions of genes frequently mutated in melanocytic neoplasms in a cohort of biopsy specimens of DN. The diagnosis was defined by histopathology, including only DNs deemed to have sufficient tumor cell content for analysis. Seventy-nine DN samples from patients aged 9 to 87 years were successfully sequenced, using a custom targeted DNA sequencing approach of a panel of known melanoma-associated genes. Only cases with at least one pathogenic mutation in any of the regions of interest were included in subsequent analyses (Table S2, Fig. S1A, and Supplementary Methods), as we could not rule out that cases without detectable mutations had insufficient tumor cellularity or sequencing coverage. In 74 cases (94.9%), a single mutation in the MAPK pathway was detected (Fig. 1A). BRAF V600E was the most common alteration (*n* = 51; 64.6%), and the average age of patients whose nevi carried this mutation was lower than that of those with other alterations (42.5 vs. 61.4 years, *P* < 0.001; Figs. 1B and S1B). When present, BRAF V600E was the only mutation identified. The melanocytic nevi without BRAF V600E mutation typically had alterative MAPK pathway activating alterations such as in NRAS and KRAS as well as NF1 and KIT. In 32.1% (9 of 28) of them, more than one pathogenic mutation was identified, with TPMs being the most frequent additional mutation (8 of 9; 88.9%; Figs. 1A and S1). In individual DNs, we also found rare pathogenic mutations in *SF3B1*, *TET2*, *TP53*, and *CDKN2A* (Fig. 1A). Overall, the mutations found were in agreement with the mutation spectrum identified in previous studies of DN and melanoma (11, 12, 25).

### Telomeres in DN with BRAF mutations are on average longer than with other MAPK drivers

**Fig. 2.**
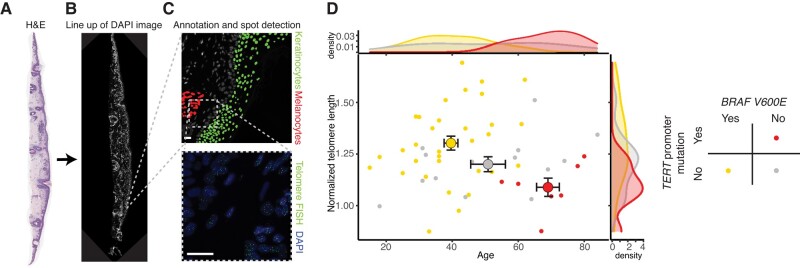
BRAFV600E mutant DN has longer telomeres than those without. Workflow for hybridization signal detection and identification of melanocytes and keratinocytes for quantitative telomere length analysis: A) Scanning magnification of dysplastic nevus stained with hematoxylin and eosin. B) Composite DAPI image of the same nevus. C) Annotated DAPI image with melanocyte and keratinocyte population and their respective hybridization signals. D) Scatter plot and density distribution of age and median normalized telomere lengths of each nevus (small, filled circles) and group means ± SE for each genotype (large, outlined circles).

Since TPMs were exclusively detectable in samples from older patients (>54 years), we hypothesized that TPMs were selected because of age-related telomere shortening. To address the question of whether cells in DN with TPMs differed in the number of divisions they had undergone, we measured the relative telomere length of melanocytes in sections of the same DN samples. Comparisons of the telomere length differences in tissues from different individuals have to take into account the heterogeneity of telomere length within the human population. To overcome this challenge, we determined the telomere length of the melanocytes in the lesion relative to the surrounding tissue keratinocytes by developing a custom hybridization and image analysis pipeline (Fig. 2A–C, Supplementary Methods). By normalizing the median intensity of telomeric signals from neoplastic melanocytes to that of the keratinocytes from the same section, we derived a “normalized telomere length” measurement, a procedure which largely eliminated variation in signal intensity between consecutive sections of cases and between individuals (Fig. S2C–H). We found that melanocytes in DN with TPMs had shorter telomeres than DN without TPMs (Fig. 2D), supporting the notion that TPMs were selected once telomeres had eroded. Grouping the data by driver mutation, we found that DN with BRAF V600E mutations had longer telomeres, whereas the DN with other driver mutations fell into two categories: (i) those with TPMs and very short telomeres, and (ii) those without TPMs and with telomeres of intermediate length (*P* = 0.008, ANOVA; Figs. 2D and S3). To compare the telomere lengths of DN to that of melanoma samples, we used melanoma samples known to carry TPMs from a prior study (19). We rank-ordered the telomere length distributions in the DN and compared them with those in the melanoma samples (Fig. S3C). We found that telomeres in DN with TPMs were of similar length or slightly longer than those in melanomas. In contrast, DN with BRAF V600E mutations had longer telomeres, indicating that these cells resulted from fewer divisions after tumor initiation than those in their counterparts driven by other MAPK mutations (Fig. 2D). We concluded that DN with driver mutations other than BRAF V600E had undergone enough rounds of cell divisions for telomeres to have become eroded. In this context, TPMs were selected for in a subgroup of DN and could be placed in a proliferative trajectory closer to melanoma than samples without TPMs.

### DN with BRAF V600E mutations are histopathologically distinct and arrest with strong p16 induction

**Fig. 3.**
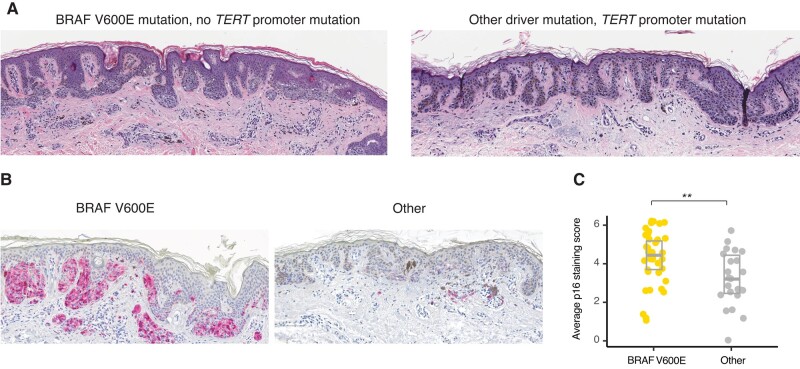
DN with BRAF V600E mutation display higher levels of p16 expression by immunohistochemistry. A) Photomicrographs of hematoxylin and eosin–stained DN with a BRAF V600E displaying a nested growth pattern (left) and without a BRAF V600E mutation and with a *TERT* promoter mutation displaying a lentiginous growth pattern with marked solar elastosis (right). B) Immunohistochemistry for p16 of a dysplastic nevus with (left panel) and without (right panel) BRAF V600E mutation. The right nevus also harbored a *TERT* promoter mutation. C) Jitter plot with boxplot of average p16 immunoreactivity score of DN (*n* = 61, *P* = 0.005, Wilcoxon rank sum test).

We then asked whether our cohort histopathologically recapitulates the differences, which have been observed in other cohorts dependent on the detected driver mutations. Independent and blinded scoring by two histopathologists revealed that the neoplastic melanocytes in DN with BRAF V600E mutations had a more nested distribution, with a larger proportion of cells in the dermis, whereas the other DN frequently had a lentiginous growth pattern and more subjacent solar elastosis (Figs. 3A and S4C). These results demonstrated that BRAF V600E DN presented a histopathologically distinct subgroup of DN in our cohort, consistent with observations made in melanomas (29) and melanocytic nevi (14). Additionally, DN without BRAF V600E mutations were more frequently classified as neoplasms of uncertain behavior (ICD-10 D48.5) at the time of diagnosis (9 of 28; 34% vs. 5 of 51; 9.8%, *P* = 0.028; Fisher's exact test) than those with the mutation. Among DN with driver mutations other than BRAF V600E, DN with TPMs also had more frequent D48.5 codes compared with those without (6 of 9; 67% vs. 8 of 70; 11.4%, *P* < 0.001, Fisher's exact test), indicating that they were histopathologically more atypical.

Based on these findings, we tested the prediction that DN with driver mutations other than BRAF V600E also expressed less of the cyclin-dependent kinase inhibitor and tumor suppressor p16, because they are not arrested via OIS, and thus progressively shorten their telomeres. To test this, we evaluated the status of p16 in our DN cohort. We scored nuclear and cytoplasmic p16 immunostaining in DN sections. We found that DN with BRAF V600E expressed higher levels of p16 than those with other driver mutations (*P* = 0.005, Kruskal–Wallis rank sum test, Figs. 3 and S4A). This difference was not dependent on the TPM status (Fig. S4B).

### Oncogenic driver mutations correlate with the mechanism of cellular immortalization in advanced melanoma

**Fig. 4.**
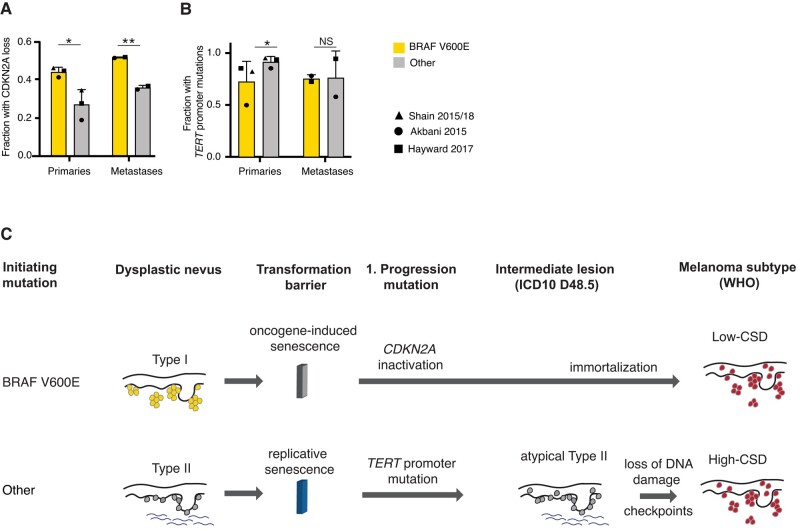
In primary melanomas with BRAF V600E mutations, biallelic inactivations of *CDKN2A* are more common and *TERT* promoter mutations are less common. Bar graph of the frequency of A) biallelic *CDKN2A* inactivation (melanoma *P* = 0.027, metastasis *P* = 0.0076, Fisher's exact test) and B) *TERT* promoter mutations (melanoma *P* = 0.024, metastasis *P* = NS, Fisher's exact test) in primary metastases and melanoma metastases patients with and without BRAF V600E mutations. Bar graphs show mean and standard deviation of fraction of patients. Value of each individual study indicated by symbols. C) Model of the evolutionary pathway of melanoma formation from the two proposed types of DN: The pathway to low-CSD melanomas (top) begins with a BRAF V600E mutation in a telomerase-negative melanocyte, which clonally expands and forms a nevus. Cell proliferation is halted by the induction of OIS, which arrests cells before they exhaust their telomeres. Transition to melanoma requires escape from OIS, which, as the genomic data from primary melanomas indicate, frequently involves disruption of the *CDKN2A* locus (11, 15). The resulting loss of cyclin-dependent kinase inhibitors p16/INK4A p14/ARF and often p15/INK4B (30) allows cells to resume proliferation, continue to shorten their telomeres, which ultimately selects for *TPMs* after formation of the primary melanoma. The pathway to high-CSD melanomas (bottom) begins with MAPK pathway mutations other than BRAF V600E (11, 31). Nevi with these mutations have a more lentiginous growth pattern and more solar elastosis. In nevi with these mutations, OIS is not triggered, and replicative lifespan constitutes the first transformation barrier. Some DN overcome this barrier by means of *TERT* promoter mutations and assume a more atypical histology. *CDKN2A* and equivalent mutations arise later during progression, *after TPMs* become prevalent.

Our observations revealed that DN with BRAF V600E showed features distinct from DN with other driver mutations, including longer telomere length and higher p16 expression. From this, we hypothesized that they would select subsequent mutations in a different sequential order, specifically melanomas arising from BRAF V600E mutant precursors would first have to override the OIS checkpoint. To test the predictions of such an orthogonal senescence pathway model, we analyzed the available melanoma datasets for the co-occurrence of cell-cycle checkpoint inactivating mutations, which are required to overcome OIS (11, 12, 15, 25). Primary melanomas with BRAF V600E mutations indeed had more frequent inactivating mutations of the *CDKN2A* gene (45.2 vs. 27.7%, *P* = 0.027, Fisher's exact test), consistent with early corruption of critical cell-cycle checkpoint components triggered by OIS (Fig. 4A). Furthermore, primary melanomas with BRAF V600E mutations had fewer TPMs than those without (77.8 vs. 93.8%, *P* = 0.024, Fisher's exact test). This difference disappeared in metastases, indicating that TPMs arise after CDKN2A inactivation in these melanomas (Fig. 4B). Similar results were obtained when less frequent mutations of the G1/S checkpoint at the level of *CDK4* or *RB1* were also included in the analysis (Fig. S5). These findings indicated that in melanomas with BRAF V600E mutations, inactivation of the G1/S checkpoint typically arises before immortalizing mutations. In melanomas with other driver mutations, TPMs arose first whereas cell-cycle checkpoint mutations emerged later.

## Discussion

By molecularly and genetically characterizing a prospectively collected set of DN, we found that DN, as defined by histopathologic features, are heterogeneous and can be divided into two main groups. Type I DN that are characterized by BRAF V600E mutations as the sole aberration and have an earlier age of onset. Their telomeres are long and their p16 expressions are similar to those of commonly acquired nevi, in keeping with the concept that they are predominantly kept in check by OIS (5). Type II DN are characterized by driver mutations other than BRAF V600E and are genetically more diverse. They have shortened telomeres, lower p16 expression levels, and frequently harbor TPMs, implicating RS as the first transformation barrier. These type II DN also have a more lentiginous growth pattern, whereas type I DN have a more nested growth pattern. Type II DN, as proposed here, partially overlap with the concept of *lentiginous nevus of the elderly*, claimed to have an elevated risk of progression to melanoma (32).

The categories of types I and II DN share key characteristics with the two main types of cutaneous melanomas defined in the current WHO Classification of Skin Tumors (33). Specifically, type I DN share frequent BRAF V600E mutations, a younger age, a more nested growth pattern, and a lower degree of solar elastosis with melanomas on skin with low cumulative sun damage (CSD) (29). In contrast, type II DN are similar to high-CSD melanomas in that they lack BRAF V600E mutations, affect older individuals, have more solar elastosis, and have a lentiginous growth pattern (34–36). Therefore, our data suggest that types I and II DN reside on two separate evolutionary trajectories, one leading to low-CSD melanoma and the other to high-CSD melanoma, with disparate phasing of transformation barriers. On the way to low-CSD melanoma, cells first encounter OIS, which is overcome by loss of CDKN2A and subsequently leads to telomere shortening followed by immortalization. The path to high-CSD melanoma would follow a different trajectory where cells continue proliferating until they face RS, which can be overcome by the acquisition of a TPM and only subsequently lose or down-regulate CDKN2A. A model illustrating the progression along the two pathways is shown in Fig. 4C.

What could be the molecular underpinnings of the two different groups of DN? The finding that telomeres remain long in DN in younger patients, which mostly have BRAF V600E mutations, suggesting that telomeres shorten differently dependent on the driver mutation. It has been shown that different mutations in the MAPK pathway activate the pathway to different degrees (13) and that high levels of activation can trigger OIS (37). Based on our observations, we conclude that the differences in telomere length in DN are the result of the differential activation of OIS and RS depending on the initiating driver mutation and represent two orthogonal senescence mechanisms limiting the progression of DN. Whether these two pathways operate completely independent of each other or have overlapping downstream will require more mechanistic studies in vitro and in vivo as suggested by Ruiz-Vega et al. (38) and reviewed in Gorgoulis et al. (39). However, an alternative explanation could be that the cell state of the melanocyte, in which the driver mutation arises, may also affect the fate of the nascent neoplasm and influence which tumor suppressor mechanisms become engaged, explaining the observed differences in telomere length and p16 accumulation (40, 41).

Genetic population studies of telomere length have correlated a longer constitutional telomere length to an increased cancer incidence rate (42). This observation is supported by rare familial melanoma mutations such as a TPM (43) or mutations in shelterin proteins POT1 and TIN2 that elongate telomeres (44–46). This effect of longer telomeres is likely because neoplasms, which are kept in check by RS, can grow to larger sizes if the constitutional telomere length is longer. At this stage of melanoma development, it therefore seems that this cancer-promoting effect is not caused by genomic instability driven by short telomeres but by the increased proliferative capacity of cells with longer telomeres. Thus, the potential number of partially transformed cells arising from cell divisions increases and with that also the number of cells, that are at risk of acquiring mutations before telomere shortening limits their proliferation (47). We show that in addition to these consequences of constitutional telomere length, the telomere length changes following the acquisition of cancer-initiating mutations impact the course of cancer development: A key aspect of our study is that we normalize for constitutional the telomere length using noncancerous cells as a reference. The analysis of relative telomere length in DN suggests that the observed length is the result of the differential activation of OIS and RS depending on the initiating driver mutation and that the two orthogonal senescence mechanisms limit the progression of DN. This measurement of relative telomere length supplements our characterization of the mutation status, the histopathology, and the p16 status and enhances our understanding of the differences between types I and II DN as an early-stage hyperplasia. Thus, our work provides a roadmap to determine whether early lesions in other cancer types that frequently have a similar genetic progression—an initial MAPK oncogenic driver mutation, including BRAF V600E, followed by a TPM—can similarly be classified into two groups that differentially engage OIS and RS.

## Materials and methods

### Patients

We prospectively collected DN based on histopathological features including a broad silhouette with lateral extension of the junctional component, eosinophilic or lamellar fibroplasia of the papillary dermis, or random cytologic atypia (48). Only lesions deemed to have sufficient tumor cell content for microdissection were included in the subsequent analyses. All lesions had received ICD10 codes for either *melanocytic nevus* (D22, *n* = 65) or *neoplasm of uncertain behavior of the skin* (D48.5, *n* = 14) at diagnosis.

### Next-generation sequencing

DNA was extracted from microdissected sections, and libraries were constructed for targeted next-generation sequencing of 80 genes implicated in melanocytic neoplasia (Table S1), using previously reported methods of analysis (11). Only cases containing at least one pathogenic mutation in any of the targeted genes were included for further analysis, as we could not rule out that cases without any detectable mutations were false negatives due to insufficient tumor cell content (details in Supplementary Methods).

### Telomere length measurement

We performed quantitative fluorescence in situ hybridization for telomeric sequences as described (19). Hybridized tissue sections were scanned at 20× magnification by structured illumination microscopy on a Rebus Biosystems synthetic aperture optics custom microscope (49, 50). Images were spatially registered with hematoxylin and eosin–stained sections to assist in identifying neoplastic melanocytes or epidermal keratinocytes (Fig. 2A–C) and analyzed without knowledge about the genotype of each case. Pixel intensity and area of individual hybridization signals were quantified using custom software (Rebus Biosystems). To mitigate the effects of inter-individual telomere length variation and varying hybridization efficiencies, we normalized the median signal intensity from neoplastic melanocytes to that of keratinocytes from the same section and derived a “normalized telomere length” measurement (Fig. S2, details in Supplementary Methods).

### Histomorphology assessment and immunohistochemistry

Two dermatopathologists independently reviewed all cases and semi-quantitatively scored solar elastosis, the proportion of melanocytes in the dermis, and degree of nest formation of melanocytes, as previously described (29). Solar elastosis was dichotomized into high (score ≥2) or low (score <2). The relative proportion of neoplastic melanocytes in the dermis, and their degree of nesting was assessed at scanning magnification and quantified as: 1: <25%; 2: 25–50%; or 3: > 50%. Interobserver agreement was assessed by a weighted kappa score and ranged from 0.48 to 0.86. Immunohistochemistry was performed on a Leica Bond instrument using a p16 antibody from Bio SB (Clone: 16P04, JC2) and the Leica Refine Red detection system. Immunoreactivity was scored blinded by two dermatopathologists separately for nuclear (0: no staining; 1: <25%; 2: 25–75%; and 3: >75% positive nuclei) and cytoplasmic (0: for no; 1: weak; 2: moderate; and 3: strong) immunoreactivity. Both scores were added, and the two observers’ means used for statistical analysis.

### Re-analysis of existent melanoma datasets

We analyzed processed mutation data from of 512 cutaneous melanomas from the following publicly available datasets: Akbani et al. (The Cancer Genome Atlas (TCGA)) (12), Hayward et al. (25), and Shain et al. (11, 15) (see details in Supplementary Methods).

### Statistical analysis

We used the Welch t test for two-way comparisons of continuous variables (age and telomere length), ANOVA for comparisons of multiple groups followed by a post hoc Tukey's Honestly Significant Difference (HSD) test, the Wilcoxon rank sum test, and Kruskal–Wallis test for ordinal variables (nesting, dermal fraction, and p16 score), and Fisher's exact test for dichotomous variables (solar elastosis, ICD-10 code, *CDKN2A* inactivation, checkpoint loss, TPM). Statistical tests and significance levels (*P < 0.05, **P < 0.01, ***P < 0.001) are indicated in the figure legends.

## Supplementary Material

pgae041_Supplementary_Data

## Data Availability

Sequencing data were deposited at in the Sequence Read Archive (SRA) under the accession number: PRJNA777884.

## Preprints

This manuscript was posted on bioRxiv: https://doi.org/10.1101/2023.07.14.548818.
