# Supplementary material for: Distinct senescence mechanisms restrain progression of dysplastic nevi
Source: PNAS Nexus. 2024 Feb 15;3(2):pgae041. doi: 10.1093/pnasnexus/pgae041 (PMC10873501; doi:10.1093/pnasnexus/pgae041)
Supplement: pgae041_Supplementary_Data [file pgae041_supplementary_data.zip › PNASNEXUS-PNASNEXUS-2023-00943R-s05.pdf]

Supporting Information for

Distinct senescence mechanisms restrain progression of dysplastic nevi.

Franziska K Lorbeer[1], Gabrielle Rieser[1], Aditya Goel[1], Meng Wang[2], Areum Oh[3], Iwei Yeh[2,4,5], Boris C Bastian[2,4,5] *, Dirk Hockemeyer[1,6,7] *

[1] Department of Molecular and Cell Biology, University of California, Berkeley, Berkeley, CA 94720, USA

[2] Department of Dermatology, University of California, San Francisco, San Francisco, CA, USA

[3] Rebus Biosystems, Santa Clara, California

[4] Department of Pathology, University of California, San Francisco, San Francisco, CA, USA

[5] Helen Diller Family Comprehensive Cancer Center, University of California, San Francisco, San Francisco, CA, USA

[6] Chan Zuckerberg Biohub, San Francisco, CA, 94158, USA

[7] Innovative Genomics Institute, University of California, Berkeley, CA, 94720

* Correspondence to: Boris Bastian, *boris.bastian@ucsf.edu* and Dirk Hockemeyer, *hockemeyer@berkeley.edu*

This PDF file includes:

Supporting text
Figures S1 to S5
Tables S1 to S4
SI References

Supporting Information Text

Supplemental Methods

Next-generation sequencing

After dissection and library construction libraries were pooled and captured with custom-designed bait libraries (xGen Lockdown probes, Integrated DNA Technologies, Coralville, Iowa) targeting the coding region of 80 genes known or suspected to be involved in the pathogenesis of cutaneous neoplasia and the promoter region of the *TERT* gene (Table S1). Sequencing was performed as paired-end, 100 base long reads on an Illumina HiSeq 4000 instrument (Illumina, San Diego, CA). The reads were aligned to the human reference sequence UCSC build hg19 (NCBI build 37), using BWA-MEM 0.7.131.(1) Variant calling was performed with Freebayes 0.9.20 and Unified Genotyper GATK.(2, 3) Variant annotation was performed with Annovar.(4) Copy number changes were called using CNVkit.(5)

Telomere length measurement

We performed quantitative fluorescence in situ hybridization (qFISH) using a Cy3-labeled probe for telomeric sequences as described previously.(6) Hybridizations were performed in three randomized batches of samples. We scanned tissue sections counterstained with 4,6-diamino-2-phenylindole (DAPI) using structured illumination microscopy at 20x magnification using an *Esper* single cell multi-omics platform (Previously named StellarVision, Rebus Biosystems, Inc., Santa Clara, CA) based on Synthetic Aperture Optics, a structured illumination microscopy method.(7, 8) In Synthetic Aperture Optics imaging, the sample was illuminated by a series of 12 high resolution light patterns formed by the interference of laser beams and the resulting series of low resolution images ("raw images"), which were then processed by *Esper* system's on-board software ("*Esper Process*") to generate a single high resolution image ("Synthetic Aperture Optics image"). This microscope allowed for an optical imaging resolution of a conventional 60x or 100x oil immersion microscopy at a low magnification and low numerical aperture air lens (20x/0.45NA), which offers more than an order of magnitude improvement in field of view, depth of field, and working distance compared to oil immersion lens. Based on this approach, we were able to image the entire tissue section with high resolution to measure the location and the intensity of telomere signals for each individual cell. Fluorescent spots representing telomere signals were detected by *Esper Process*, first by finding all local intensity peaks from the reconstructed Synthetic Aperture Optics image, followed by intensity thresholding to filter out the background. For each of the remaining spots, corresponding pixel location in the raw images was identified and the average value and the modulation strength of the series of 12 intensities of the pixel were calculated, based on which false positive spots were further eliminated.

Individual images were stitched together and spatially registered with a digital image of a hematoxylin and eosin (HE) stained section to assist in identifying regions of interest containing neoplastic melanocytes or epidermal keratinocytes (Fig. 2A-C). Individual nuclei were segmented based on the DAPI image using the implementation of Huang's fuzzy thresholding in Fiji to turn DAPI images into binary images and the segmented nuclei were subsequently color coded as melanocytes or keratinocytes using morphologic and positional information from the HE images without knowledge about the genotype of each case (Fig. 2B, Fig. S2A). The minimum number of melanocytes quantified was 284 with an average number of 1265.4 melanocytes evaluated per case.

Based on their position, the telomeric signals were then assigned to individual keratinocytes and melanocytes and the median telomeric signal per cell was calculated (Fig. S2A-B). Telomere length varies among individuals, partially dependent on age and constitutional telomere length.(9, 10) Additionally, the intensity of hybridization signals can vary depending on tissue fixation, hybridization conditions, and batch effects (Fig. S2B,D). To mitigate both effects on the telomere length measurement, we normalized the median signal intensity from neoplastic melanocytes to that of the keratinocytes from the same section and derived a "normalized telomere length" measurement. This procedure largely eliminated variation in hybridization intensity between consecutive sections of cases (Fig. S2C-H) and eliminated inter-individual variation of telomere length. Data analysis was performed in R and plots generated with ggplot2.

Re- analysis of existent melanoma datasets

We analyzed processed mutation data from of 512 cutaneous melanomas from the following publicly available datasets: Akbani et al. (TCGA)(11), Hayward et al.(12) and Shain et al.(13, 14). Copy number data were obtained from the supplementary material(12–14), from the Genomic Data Commons data portal of the National Cancer Institute(11), or recalculated from the BAM alignment files using FACETS(15, 16). Samples that were from cell lines, could not be unequivocally classified as primary or metastasis, or had insufficient tumor cell content to derive copy number variation profiles were excluded, leaving 482 cases for further analyses.

Samples that had either inactivation of *CDKN2A* or *RB1* or had activation of CDK4 were considered to have an inactivated G1/S cell cycle checkpoint. *CDKN2A* and *RB1* were considered inactivated, if their loci displayed a deep deletion, or their coding sequences had a pathogenic mutation accompanied by either a shallow deletion, loss of heterozygosity, or an additional pathogenic mutation. Four samples with somatic mutations of unknown significance of *CDKN2A* (F90L, L65LL insertion, M52R and A57S) were excluded from the analyses. Activation of CDK4 was defined by the presence of a known activating mutation or amplification of the *CDK4* locus. *TERT* promoter mutation status could be derived from 263 samples that had sufficient sequencing coverage of the *TERT* promoter.(13)

Figure S1

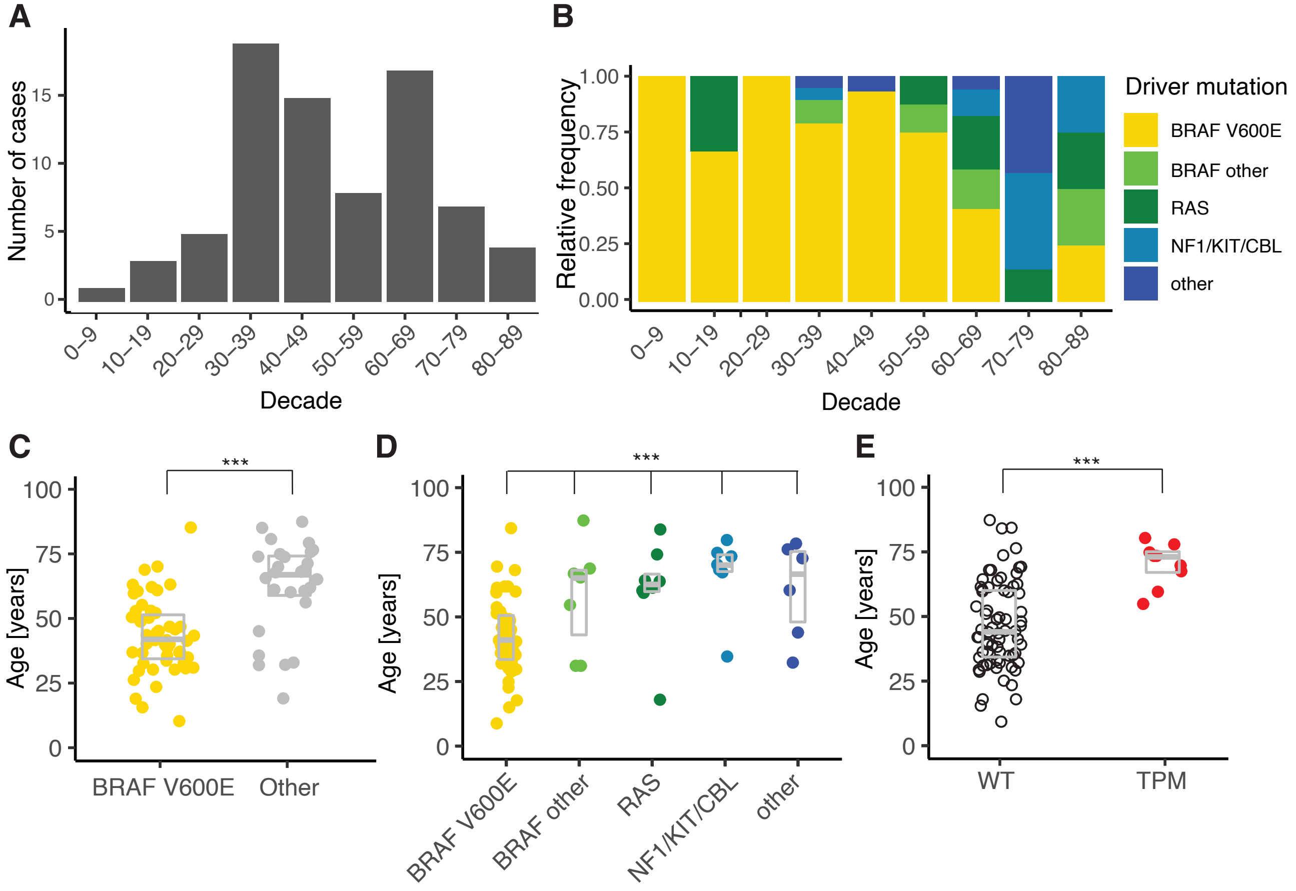

Fig. S1: Age distribution of dysplastic nevi, their identified driver mutations and histopathological analysis. (A) Age distribution of the sequenced dysplastic nevi. (B) Relative frequency of driver mutations per age decade. (C) Patient age by BRAF V600E status (*** p<0.001, Welch's t-test), (D) Individual subset of MAP-kinase pathway mutations (p<0.001, ANOVA), (E) *TERT* promoter mutation (TPM) status (p<0.001, Welch's t-test).

Figure S2

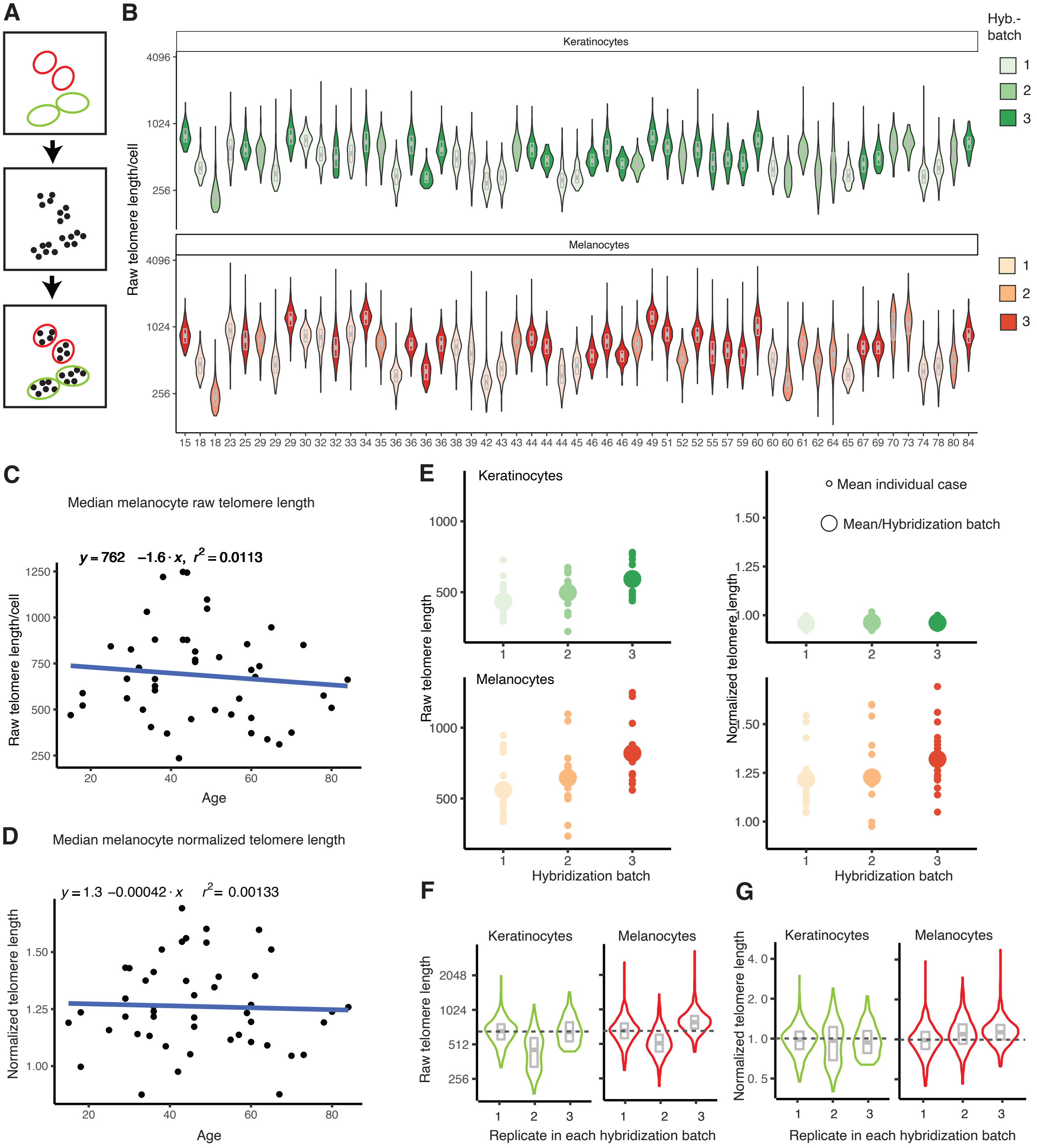

Fig. S2: Normalization of melanocyte telomere length measurements to adjacent keratinocytes minimizes batch effects. (A) Schematic overview of the process of combining cell annotation information with hybridization signals to extract cell type-specific telomere length measurements. (B) Violin plots with inset boxplots showing median and quartiles of telomeric signals before normalization (red = melanocytes, green = keratinocytes, shades indicate staining batches). Scatter plot of raw (C) and normalized (D) median telomere length measurements of neoplastic melanocytes and patient age with linear regression line. (E) Median telomeric signal of melanocytes and keratinocytes in each nevus (small circle) and the average of all cases (big circle) for the three hybridization batches before (raw telomere length) and after normalization (relative telomere length). Violin plots with inset boxplots showing median and quartiles of raw (F) and normalized (G) telomeric signals of a dysplastic nevus stained in all three hybridization batches.

Figure S3

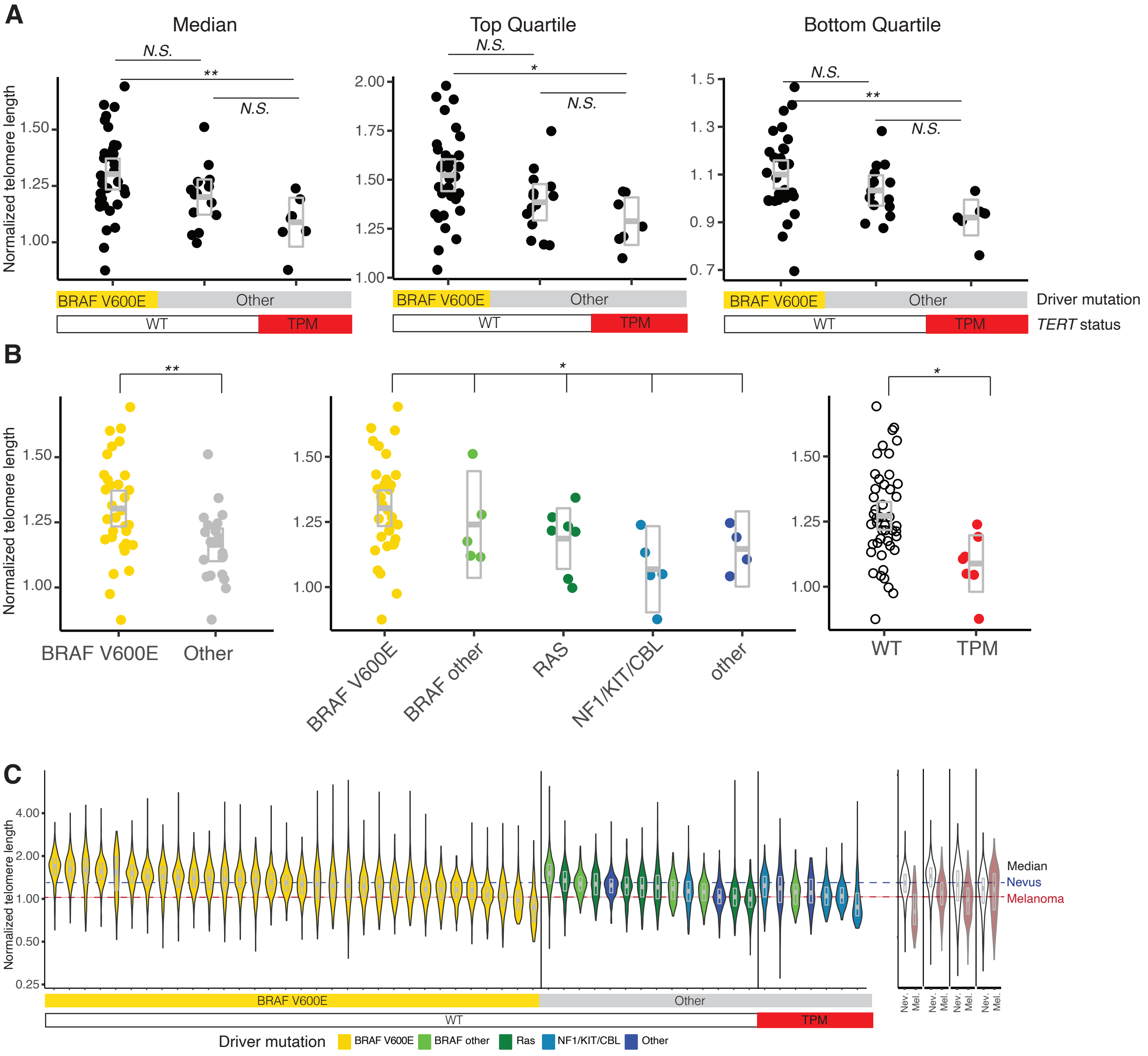

Fig. S3: Telomere length in dysplastic nevi varies depending on MAP-kinase pathway driver and *TERT* promoter mutation status. (A) Jitter plots with inset boxplots of median, top and bottom quartile telomere lengths show that the cells of dysplastic nevi with BRAF V600E mutation tend to have longer telomeres over the entire distribution of telomere lengths within a given lesion (Median: p = 0.008, ANOVA, p = 0.011, Tukey multiple comparisons, Top quartile: p = 0.010, ANOVA, p = 0.019, Tukey HSD, Bottom quartile: p = 0.012, ANOVA, p = 0.011, Tukey HSD). (B) Jitter plots with inset boxplots of normalized telomere length compared by BRAF V600E mutation status (left panel, p = 0.006, Welch's t-test), by groups of MAP-kinase driver mutations (middle panel, p = 0.039, ANOVA), and by *TERT* promoter mutation status (right panel, p = 0.013, Welch's t-test).(C) Normalized telomere length of the successfully analyzed cases (n = 53) by BRAF V600E and TERT promoter mutation (TPM) status. The median telomere length of four matched adjacent nevus and melanoma cases shown on the right is indicated by the dashed lines for comparison.

Figure S4

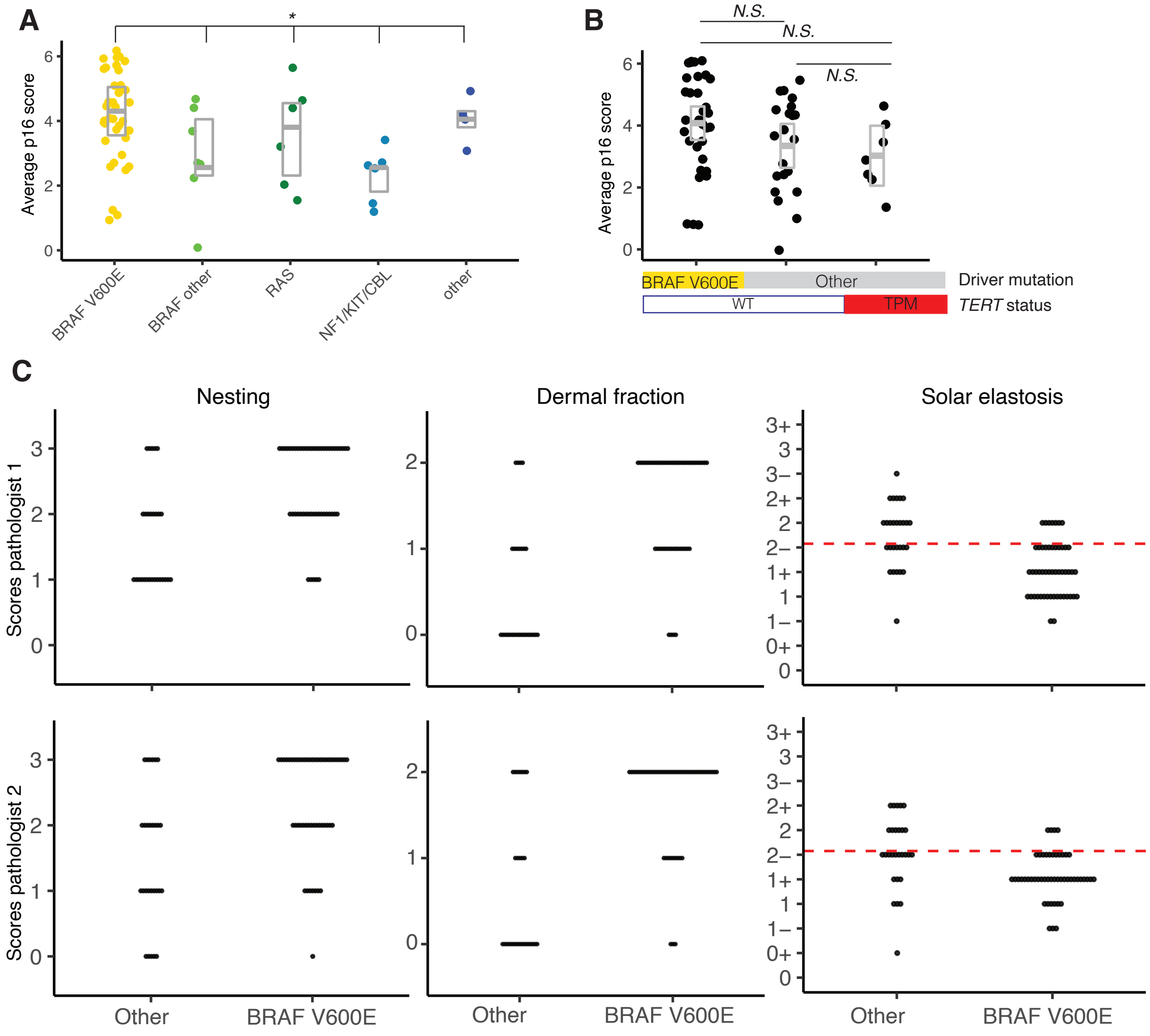

Fig. S4 The p16 score of BRAF V600E mutant nevi is higher than of those with other driver mutations: (A) Jitter plot and boxplots of the average p16 score for all driver mutations (n = 61, p = 0.02, Kruskal-Wallis test) (B) Jitter plot and boxplots showing the average p16 score of all cases separated by BRAF V600E and *TERT* promoter mutation status(WT/TPM) status (n= 61, p , Kruskal-Wallis test). (C) Histopathological analysis of dysplastic nevi with BRAF V600E and other driver mutations scored by two pathologists: Nesting (Wilcoxon rank sum test, pathologist 1: p<0.001, pathologist 2: p<0.001, weighted kappa concordance coefficient: 0.51), Dermal fraction (Wilcoxon rank sum test, pathologist 1: p<0.001, pathologist 2: p = 0.005, weighted kappa concordance coefficient: 0.86), Solar elastosis (Fisher's exact test High >=2, Low <2 (dashed red line), pathologist 1: p < 0.001, pathologist 2: p = 0.003, weighted kappa concordance coefficient: 0.30).

Figure S5

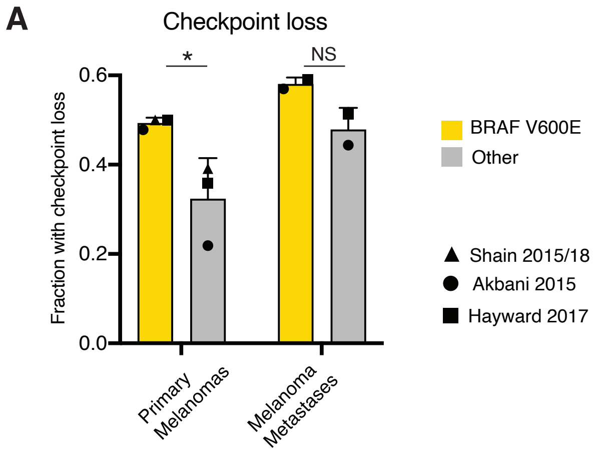

Fig. S5 G1/S ceckpoint loss is more common in primary melanoma with driver mutations other than BRAF V600E: (A) Bar graphs showing the mean and standard deviation of the fraction of patients with G1/S checkpoint loss by BRAF V600E mutation status in primary melanomas and metastatic melanomas. Averages in individual studies are represented by different symbols (melanoma p = 0.042, metastasis N.S., Fisher's exact test).

Dataset S1

Genes included in the sequencing panel

Dataset S2

Demographic and clinical information of all samples

Dataset S3

Summary of sequencing data of all samples

Dataset S4

Telomere length and p16 immunohistochemistry data

SI References

1. H. Li, R. Durbin, Fast and accurate short read alignment with Burrows–Wheeler transform. *Bioinformatics* **25**, 1754–1760 (2009).

2. E. Garrison, G. Marth, Haplotype-based variant detection from short-read sequencing. *ArXiv12073907 Q-Bio* (2012) (March 3, 2021).

3. A. McKenna, *et al.*, The Genome Analysis Toolkit: A MapReduce framework for analyzing next-generation DNA sequencing data. *Genome Res.* **20**, 1297–1303 (2010).

4. K. Wang, M. Li, H. Hakonarson, ANNOVAR: functional annotation of genetic variants from high-throughput sequencing data. *Nucleic Acids Res.* **38**, e164–e164 (2010).

5. E. Talevich, A. H. Shain, T. Botton, B. C. Bastian, CNVkit: Genome-Wide Copy Number Detection and Visualization from Targeted DNA Sequencing. *PLOS Comput. Biol.* **12**, e1004873 (2016).

6. K. Chiba, *et al.*, Mutations in the promoter of the telomerase gene TERT contribute to tumorigenesis by a two-step mechanism. *Science* **357**, 1416–1420 (2017).

7. M. G. L. Gustafsson, Surpassing the lateral resolution limit by a factor of two using structured illumination microscopy. *J. Microsc.* **198**, 82–87 (2000).

8. J. Ryu, S. S. Hong, B. K. P. Horn, D. M. Freeman, M. S. Mermelstein, Multibeam interferometric illumination as the primary source of resolution in optical microscopy. *Appl. Phys. Lett.* **88**, 171112 (2006).

9. R. C. Allsopp, *et al.*, Telomere length predicts replicative capacity of human fibroblasts. *Proc. Natl. Acad. Sci. U. S. A.* **89**, 10114–10118 (1992).

10. N. Rufer, *et al.*, Telomere Fluorescence Measurements in Granulocytes and T Lymphocyte Subsets Point to a High Turnover of Hematopoietic Stem Cells and Memory T Cells in Early Childhood. *J. Exp. Med.* **190**, 157–168 (1999).

11. R. Akbani, *et al.*, Genomic Classification of Cutaneous Melanoma. *Cell* **161**, 1681–1696 (2015).

12. N. K. Hayward, *et al.*, Whole-genome landscapes of major melanoma subtypes. *Nature* **545**, 175–180 (2017).

13. A. H. Shain, *et al.*, The Genetic Evolution of Melanoma from Precursor Lesions. *N. Engl. J. Med.* **373**, 1926–1936 (2015).

14. A. H. Shain, *et al.*, Genomic and Transcriptomic Analysis Reveals Incremental Disruption of Key Signaling Pathways during Melanoma Evolution. *Cancer Cell* **34**, 45-55.e4 (2018).

15. M. Krauthammer, *et al.*, Exome sequencing identifies recurrent mutations in NF1 and RASopathy genes in sun-exposed melanomas. *Nat. Genet.* **47**, 996–1002 (2015).

16. R. Shen, V. E. Seshan, FACETS: allele-specific copy number and clonal heterogeneity analysis tool for high-throughput DNA sequencing. *Nucleic Acids Res.* **44**, e131 (2016).
